# *Lycium ruthenicum* Murray Anthocyanins Alleviate Aging Through SIRT1/P53 Signaling Pathway

**DOI:** 10.3390/ijms26104510

**Published:** 2025-05-09

**Authors:** Jialin Liang, Zang Ga, Jiaqin Wu, Yingjie Wang, Nanjia Dongzhu, Rangzhong Qieyang, Ping Li, Sangduo Huaqian

**Affiliations:** 1 College of Ecological Environment Engineering, Qinghai University, Xining 810016, China; ys230860010522@qhu.edu.cn; 2 College of Tibetan Medicine, Qinghai University, Xining 810016, China; ys221005130849@qhu.edu.cn (Z.G.); ys231055001510@qhu.edu.cn (J.W.); 2003980002@qhu.edu.cn (N.D.); 2005980007@qhu.edu.cn (R.Q.); 3 Medical Department, Qinghai University, Xining 810016, China; wyj@qhu.edu.cn; 4 State Key Laboratory of Plateau Ecology and Agriculture, Qinghai University, Xining 810016, China

**Keywords:** *Lycium ruthenicum* Murray, anthocyanins, anti-aging, SIRT1/P53 pathway

## Abstract

Aging-related diseases have become a global health issue, with the escalating aging population leading to an increased disease incidence, placing immense pressure on individual health and society. *Lycium ruthenicum* Murray anthocyanins are hailed as the “Black Pearl of the Desert”. Anthocyanins are potent natural antioxidants that can combat oxidation, reduce inflammation, prevent cardiovascular diseases, protect the liver, and inhibit tumor cell growth. As individuals age, the accumulation of free radicals in the body accelerates aging. Antioxidants mitigate aging by neutralizing free radicals, and the anthocyanins in *Lycium ruthenicum* Murray effectively reduce oxidative damage, activate the antioxidant enzyme system, and enhance the body’s antioxidant capacity, thereby slowing the aging process. This study investigated *Lycium ruthenicum* Murray Anthocyanins’ (LRAs) anti-aging mechanisms using D-galactose-induced H9c2 cells and H_2_O_2_-treated zebrafish. LRAs increased survival rates (30.47% cells, 20.02% zebrafish), reduced ROS, Sa-β-gal, and apoptosis markers, while boosting antioxidant enzymes (SOD, CAT, GSH) and lowering MDA. It upregulated Bcl-2/SIRT1 and downregulated Bax/P53/P21/NF-κB/MAPK/TNF-α genes, with protein-level SIRT1 activation and P53/P21 suppression. The transcriptome analysis revealed a significant reduction in aging-related gene expression levels. The results demonstrated that LRAs mitigate aging through SIRT1/P53-mediated oxidative stress inhibition and apoptosis reduction, suggesting their therapeutic potential for age-related disorders.

## 1. Introduction

Global demographic trends reveal a critical challenge: while advancements in medical technology have driven population growth, aging populations now pose significant socioeconomic burdens worldwide [1]. Aging represents the comprehensive decline of various bodily functions over time. It is an inherent part of the life process and the natural pathway to an individual’s eventual death [2]. This inevitable biological progression becomes clinically consequential when exacerbated, predisposing individuals to multimorbidity, including neurodegenerative disorders [3], malignancies [4], cardiovascular diseases [5], ocular pathologies (cataracts [6], glaucoma [7]), and metabolic dysregulation (diabetes [8]), which are all major contributors to geriatric mortality and quality of life deterioration [9,10,11].

Among aging mechanisms, the Free Radical Theory is the predominant scientific consensus [12]. This theory proposes that the biological aging process is characterized by a series of degenerative changes in the organism, primarily driven by the accumulation of reactive oxygen species (ROS) generated during normal cellular metabolic processes. Excessive ROS levels cause oxidative damage to cellular components including DNA, proteins, lipids, and other macromolecules, thereby impairing physiological functions [13]. Persistent ROS accumulation further induces mitochondrial DNA (mtDNA) lesions, promotes DNA strand breaks, and contributes to mtDNA impairment. Mitochondrial dysfunction exerts pleiotropic effects on cellular homeostasis [14]. Notably, murine models with mtDNA mutations exhibit accelerated aging phenotypes [15], underscoring mitochondrial integrity as a critical longevity determinant [16]. Thus, identifying methods that mitigate aging not only serves as an effective preventive measure against aging-related diseases but also significantly reduces healthcare costs and enhances the quality of life for individuals.

*Lycium ruthenicum* Murray (Solanaceae), a congener of *Lycium barbarum*, represents a nutrient-dense economic crop endemic to northwest China (Qinghai–Tibet Plateau), bearing purple–black berries with a distinctive sweet flavor profiles [17]. Dubbed the "desert’s black pearl" and “soft gold” for its therapeutic potential, this species yields anthocyanin-rich extracts (*Lycium ruthenicum* Murray anthocyanins, LRAs) with multisystem bioactivities, including antioxidant [18], anti-inflammatory [19], anti-gouty arthritis [20], hepatoprotective [21], hypoglycemic [22], anti-obesity [23], and gut microbiota-modulating effects [24,25]. Relevant mechanistic studies have shown that LRAs exhibit multidimensional regulatory capacities: Yan et al. demonstrated that anthocyanins inhibit NF-κB signaling pathway activation through the suppression of reactive oxygen species (ROS) and enhancement of cellular antioxidant defenses, thereby ameliorating oxidative stress and inflammatory responses in acute kidney injury models [26]; Sadehi et al. validated anthocyanins’ capacity to extend milk oxidation stability and preserve unsaturated fatty acid levels during storage [27]; Wu et al. revealed that *Lycium ruthenicum* Murray berry-derived anthocyanins suppress autophagy in RAW264.7 murine macrophages via critical regulators of the AMPK-mTOR signaling axis [28]; Wei et al. experimentally established that anthocyanins from Qaidam *Lycium ruthenicum* Murray exert photoprotective effects by counteracting UVB-induced premature senescence in human skin fibroblasts (HSFs) [29]. Nevertheless, the systematic evaluation of LRAs’ anti-aging efficacy against oxidative stress-mediated senescence remains unexplored, representing a critical knowledge gap that this study addresses.

## 2. Results

### 2.1. LRAs Attenuate D-gal-Induced Cellular Senescence

**Figure 1:**
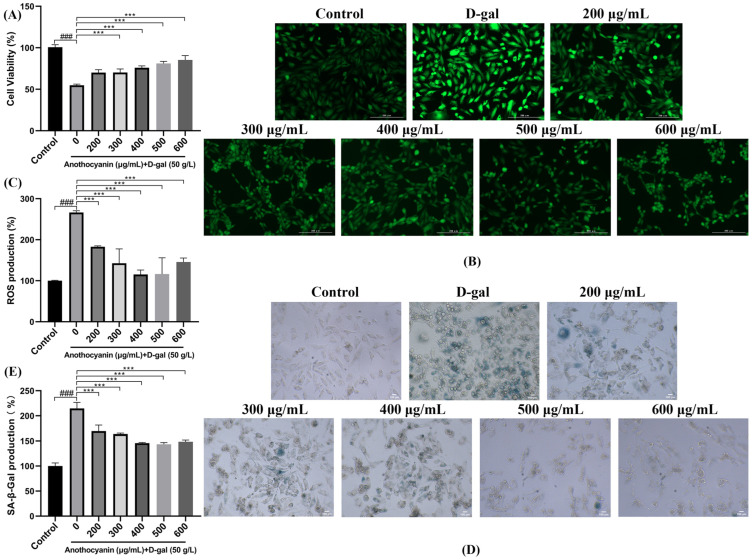
Effect of LRA on D-gal-induced senescence of H9c2 cells. (**A**) Cell viability was evaluated by CCK-8 assay; (**B**) ROS fluorescence staining (microscopic magnification: 200×); (**C**) Quantitative analysis of ROS fluorescent intensity; (**D**) SA-β-gal fluorescent staining (microscopic magnification: 200×); (**E**) Quantitative analysis of SA-β-gal fluorescence intensity; Statistical significance: ^###^ *p* < 0.001 vs. Control group; *** *p* < 0.001 vs. D-gal group.

During cellular aging, the apoptotic rate of H9c2 cardiomyocytes progressively escalated. To investigate the cytoprotective effects of LRAs, we evaluated their impact on D-galactose-induced senescent H9c2 cells through the quantitative measurement of the proliferation capacity using the CCK-8 assay according to the manufacturer’s protocols. As shown in Figure 1A, the D-gal treatment (50 g/L) reduced cell viability to 54.9% compared to the untreated control (100%, *p* < 0.001). The groups treated with LRAs (200–600 μg/mL) all showed the dose-dependent recovery of the cell survival rate (the maximum protective effect occurred at 600 μg/mL, with 78.3 ± 5.2 %, *p* < 0.001). These results indicate that LRAs can effectively inhibit cellular senescence induced by D-gal treatment in H9c2 cells.

### 2.2. LRAs Scavenge ROS and Alleviate Cell Aging

To investigate the protective effects of LRAs against D-gal-induced oxidative stress and senescence in H9c2 cells, its potential anti-aging mechanisms were evaluated and the optimal effective concentration was identified. By detecting key senescence markers such as ROS levels and SA-β-gal activity, we systematically analyzed the role of LRAs in alleviating D-gal-induced cellular damage. The DCF-DA results presented in Figure 1B indicate that, compared to the cell control group, the intracellular ROS fluorescence intensity in the D-gal treated group significantly increased. This finding suggests that D-gal exposure leads to a notable rise in intracellular oxidative stress. The elevation of ROS levels serves as a critical marker of cellular damage and oxidative stress, which are pivotal in the pathogenesis of various diseases, particularly those associated with aging. However, after 24 h of treatment with different concentrations of LRAs, the intracellular ROS fluorescence intensity was found to be lower than that of the D-gal model group. This observation implies that LRAs possess anti-aging properties and can effectively mitigate D-gal-induced aging. The ROS fluorescence intensity in the LRA treatment group was consistently lower than that in the D-gal group, further confirming LRAs’ efficacy in alleviating D-gal-induced ROS accumulation. Further quantitative analysis (Figure 1C) revealed that the ROS expression level in the D-gal group was 266.36% of that in the control group, underscoring a significant increase in oxidative stress due to D-gal treatment. In comparison, the LRA-treated group exhibited a 150.9% reduction in ROS levels compared to the D-galactose (D-gal)-induced model group (115.4% vs. 266.3% relative expression, *p <* 0.001), demonstrating the significant attenuation of oxidative stress at a 400 μg/mL LRA concentration. Collectively, these results demonstrate that LRAs can diminish oxidative stress in H9c2 cells, providing robust evidence for LRAs’ role in alleviating aging.

The SA-β-gal staining results (Figure 1D) provide crucial insights into the impact of LRAs on D-gal-induced senescence in H9c2 cells. In the D-gal group, a significant increase in the number of dark-blue-stained cells was observed, which serves as a hallmark of cellular senescence. This dark blue staining is attributed to the accumulation of senescence-associated β-galactosidase, indicating a state of advanced senescence and dysfunction in the cells. In contrast, treatment with LRAs for 24 h resulted in a significant reduction in dark blue staining, indicating that LRA treatment can reverse the D-gal-induced aging phenotype. Further quantitative results showed that the expression level in the D-gal group was 214.6% of that in the control group. At an LRA concentration of 500 μg/mL, the expression level was 143.5%, representing a 71.1% decrease compared to the D-gal group. This suggests that this concentration of LRAs exerts the strongest protective effect (Figure 1E). This concentration appears to be the optimal dose for reversing D-gal-induced cellular senescence.

### 2.3. LRAs Alleviate D-gal-Induced Apoptosis

**Figure 2:**
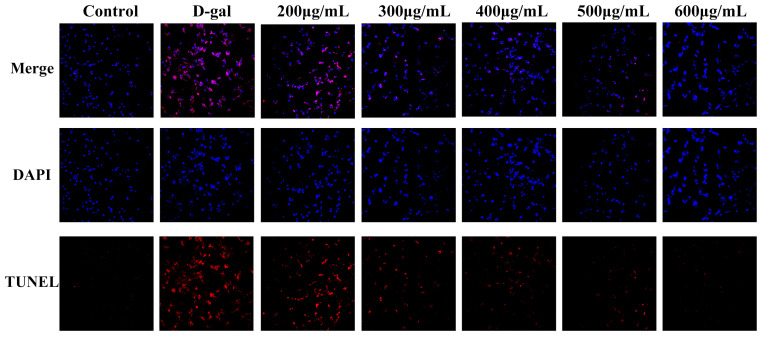
The effect of LRAs on D-galactose-induced apoptosis in H9c2 cells. TUNEL and DAPI co-staining (microscopic magnification: 100×). Red represents TUNEL, and blue represents DAPI.

To evaluate the protective effects of LRAs against D-gal-induced apoptosis and nuclear morphological damage using TUNEL/DAPI co-staining, and to elucidate their regulatory mechanisms in programmed cell death, TUNEL was primarily employed to detect cell apoptosis, also known as programmed cell death. It utilizes terminal deoxynucleotidyl transferase (TdT) to add fluorescently labeled nucleotides to the ends of DNA, rendering them detectable under a microscope. DAPI binds to DNA within the cell nucleus and emits blue fluorescence. The combined application of DAPI and TUNEL facilitates the simultaneous observation of cell apoptosis and nuclear morphological changes. The co-staining results of TUNEL and DAPI (Figure 2) indicated that, compared to the control group, the number of apoptotic cells in the D-galactose group increased, and the nuclei exhibited irregular shapes. In contrast, when compared to the D-galactose group, the number of apoptotic cells in the LRA group decreased, and the morphology of the nuclei showed partial restoration.

### 2.4. Effects of LRAs on Survival Rate and Heartbeat in Zebrafish

**Figure 3:**
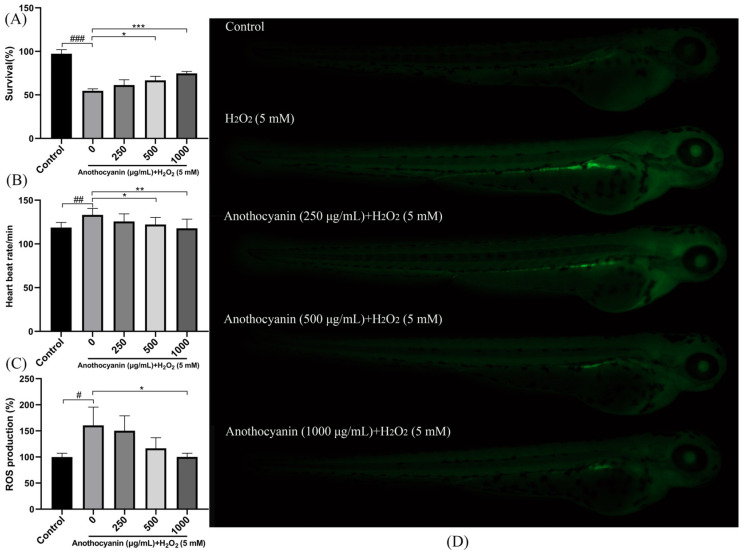
Effect of LRAs on H_2_O_2_-induced survival rate, heartbeat, and ROS production in zebrafish larvae. (**A**) The impact of LRA on zebrafish survival rate; (**B**) the impact of LRAs on zebrafish heartbeat; (**C**,**D**) effect of LRAs on H_2_O_2_-induced ROS production in zebrafish larvae; statistical significance: *^#^ p* < 0.05, *^##^ p* < 0.01, *^###^ p* < 0.001 vs. Control group; * *p* < 0.05, ** *p* < 0.01, *** *p* < 0.001 vs. H_2_O_2_ group.

To evaluate the protective effects of LRAs, zebrafish larvae were treated with H_2_O_2_. As shown in Figure 3A, the survival rate of the blank control group was 100%, whereas H_2_O_2_ treatment significantly reduced the survival rate to 54.60%. In contrast, pretreatment with 1000 μg/mL LRAs increased the survival rate to 74.66% (*p <* 0.001 vs. H_2_O_2_ group). Heartbeat analysis (Figure 3B) revealed that the blank group exhibited a baseline heart rate of 118.62 beats/min, while H_2_O_2_ treatment increased the heart rate to 133.13 beats/min. Notably, all LRA-treated groups showed significantly lower heart rates compared to the H_2_O_2_ group (*p <* 0.01). These results indicate that LRAs mitigate H_2_O_2_-induced aging in zebrafish larvae.

### 2.5. Protective Effect of LRAs on H_2_O_2_-Induced ROS Generation in Zebrafish

To evaluate the antagonistic effect of LRAs on H_2_O_2_-induced oxidative stress in zebrafish embryos, the DCF-DA fluorescence assay was employed. As shown in Figure 3D, compared with the blank group, H_2_O_2_ treatment significantly increased the ROS fluorescence intensity from 100% to 160.6%. However, after pretreatment with 1000 μg/mL LRAs, the ROS fluorescence intensity was 100%, and the ROS level significantly decreased to below that of the H_2_O_2_ group (*p <* 0.05). In addition, LRAs exhibited the dose-dependent inhibition of H_2_O_2_-induced ROS accumulation (Figure 3C).

### 2.6. Protective Effect of LRAs on H_2_O_2_-Induced Zebrafish Cell Senescence

**Figure 4:**
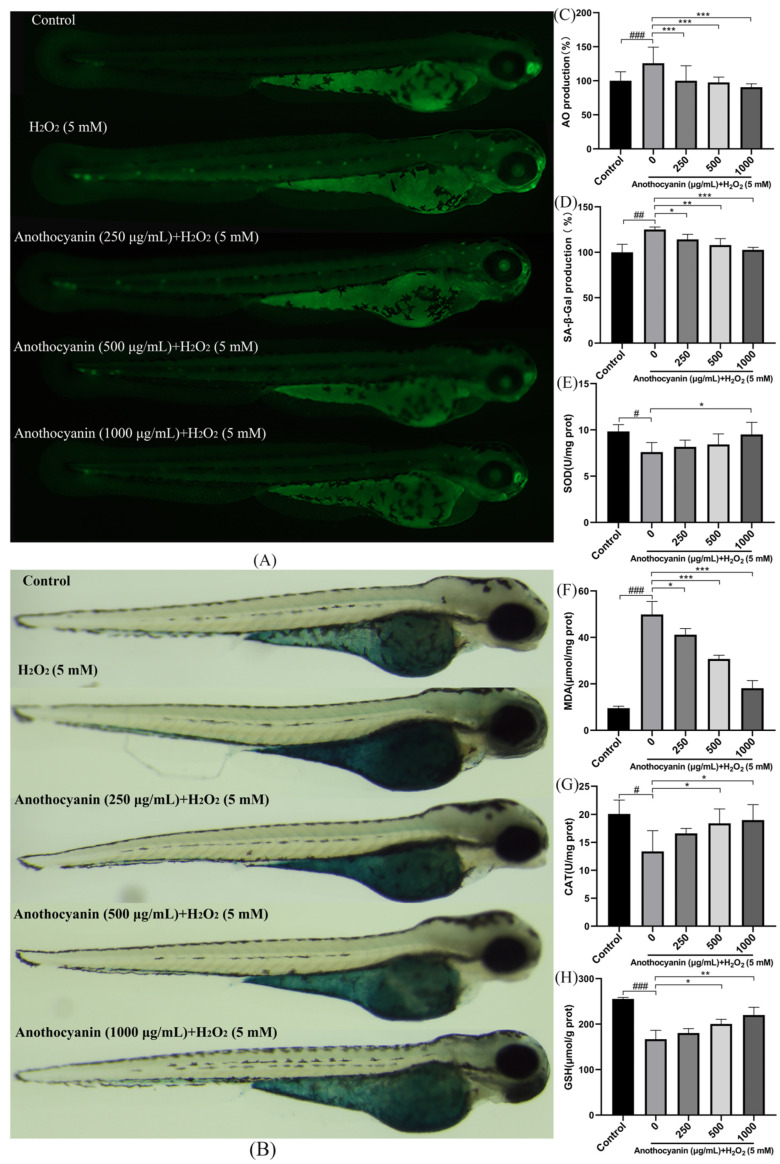
(**A**,**C**) Effect of LRA on H_2_O_2_-induced AO fluorescence in zebrafish larval; (**B**,**D**) Effect of LRA on H_2_O_2_-induced SA-β-gal activity in zebrafish larval; (**E**–**H**) Effects of LRA on oxidative stress markers: (**E**) SOD; (**F**) MDA; (**G**) CAT; (**H**) GSH; Statistical significance: ^#^ *p* < 0.05, ^##^ *p* < 0.01, ^###^ *p* < 0.001 vs. Control group; * *p* < 0.05, ** *p* < 0.01, *** *p* < 0.001 vs. H_2_O_2_ group.

We evaluated the antagonistic effects of LRAs against H_2_O_2_-induced DNA damage and cellular senescence in zebrafish using AO staining and SA-β-gal assays. AO is a fluorescent dye that can bind to both DNA and RNA. It exhibits a stronger affinity for DNA, which allows it to enter cells and bind to DNA, thereby generating a fluorescent signal. Senescent cells typically accumulate DNA damage, and acridine orange staining can effectively reveal such damage. In particular, the structure of DNA in senescent cells may undergo alterations or breakages, leading to distinct fluorescence emission patterns that assist in the identification of senescent cells. AO staining (Figure 4A) showed that the fluorescence intensity in the H_2_O_2_ group significantly increased (266.36%, *p <* 0.001). LRA pretreatment effectively reduced AO fluorescence in a concentration-dependent manner, and at a concentration of 1000 μg/mL, the expression level was only 90.6% (Figure 4C, *p <* 0.001). SA-β-gal staining further confirmed these findings: H_2_O_2_-treated zebrafish exhibited distinct blue staining in the dorsal and ventral regions, while LRA pretreatment significantly attenuated this senescence-associated signal (Figure 4B,D, *p <* 0.05).

### 2.7. Effect of LRAs on H_2_O_2_ Induced Oxidative Stress Factors in Zebrafish Larvae

Zebrafish possess a variety of antioxidant enzymes and non-enzymatic antioxidant molecules that help combat oxidative stress. As aging progresses, these antioxidant defense mechanisms may weaken, resulting in increased oxidative stress. Therefore, by measuring changes in the levels of SOD, MDA, CAT, and GSH, researchers can assess the alterations in antioxidant defense mechanisms during aging. Compared to the blank group, H_2_O_2_ treatment significantly decreased antioxidant enzyme activities (SOD, CAT and GSH) while increasing MDA levels, a marker of lipid peroxidation (*p <* 0.001). Conversely, LRA intervention restored SOD, CAT and GSH activities and reduced the MDA content in a dose-dependent manner (Figure 4E–H, *p <* 0.05. These data collectively indicate that LRAs alleviate oxidative stress and exert anti-aging effects in zebrafish.

### 2.8. Effect of LRAs on H_2_O_2_ Induced Genes Related to Zebrafish Larvae

**Figure 5:**
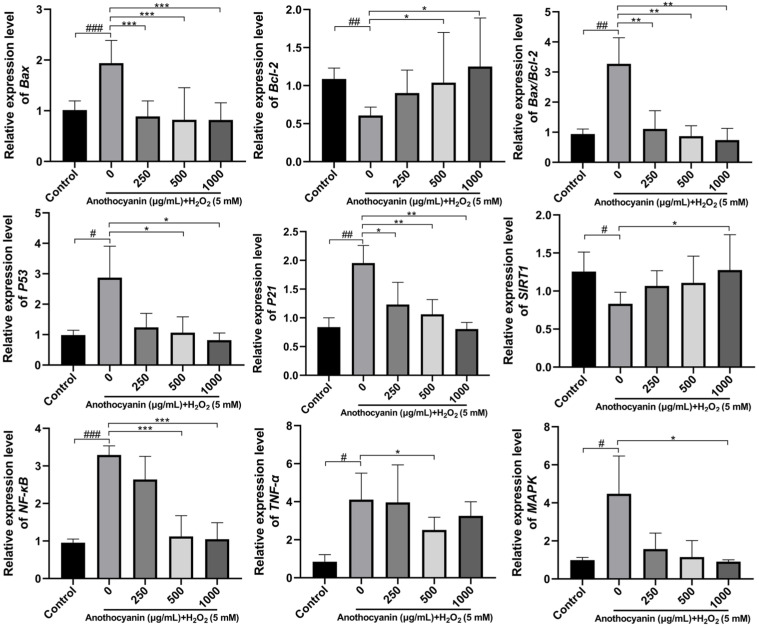
Effects of LRA on the expression of related genes in zebrafish. Statistical significance: ^#^ *p* < 0.05, ^##^ *p* < 0.01, ^###^ *p* < 0.001 vs. Control group; * *p* < 0.05, ** *p* < 0.01, *** *p* < 0.001 vs. H_2_O_2_ group.

To further investigate the anti-aging mechanism of LRAs, RT-PCR was performed to analyze key genes (Figure 5). H_2_O_2_ treatment significantly upregulated pro-apoptotic gene expression (*Bax*, *TNF-α*, *MAPK*, *NF-κB*, *P21*, *P53*) and increased the *Bax/Bcl-2* ratio, while downregulating apoptotic *Bcl-2* and *SIRT1*. LRA pretreatment reversed these effects, reducing pro-apoptotic gene expression and restoring *Bcl-2* and *SIRT1* levels (*p <* 0.05).

### 2.9. Effect of LRAs on SIRT1/P53 Signaling Pathway

**Figure 6:**
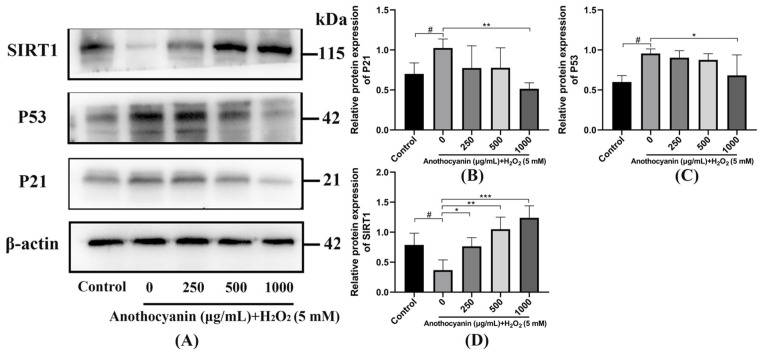
LRA alleviates aging through the SIRT1/P53 signaling pathway. (**A**) Effect of LRA on SIRT1/P53 protein; (**B**) Relative quantification of P21 protein. (**C**) Relative quantification of P53 protein. (**D**) Relative quantification of SIRT1 protein. Statistical significance: ^#^ *p* < 0.05 vs. Control group; * *p* < 0.05, ** *p* < 0.01, *** *p* < 0.001 vs. H_2_O_2_ group.

As shown in Figure 6A, H_2_O_2_ treatment significantly suppressed SIRT1 protein expression while increasing P53 and P21 levels. LRA intervention restored SIRT1 expression and reduced P53 in a dose-dependent manner. As shown in Figure 6B, in comparison to the control group, the expression level of P21 in the model group exhibited a notable increase of 32.25%. This finding indicates a substantial alteration in P21 expression, suggesting that the conditions of the model group may have impacted cellular regulation processes. On the other hand, when looking at the effects of LRAs at a concentration of 1000 μg/mL, there was a significant reduction in the expression level of the SIRT1 protein within the model group. Specifically, the SIRT1 protein expression level decreased by 50.96% in this experimental condition, highlighting the potential regulatory effects of LRAs on protein expression dynamics. Furthermore, the analysis presented in Figure 6C demonstrates that the expression level of P53 in the model group was significantly elevated by 35.68% when compared to the control group. In contrast to the model group, there was a significant decrease of 27.35% in the P53 protein expression level in the group treated with 1000 μg/mL LRAs. When compared to the control group, the SIRT1 expression level in the model group exhibited a substantial reduction of 42.03%. Furthermore, relative to the model group, the 1000 μg/mL LRA group showed a notable increase of 81.85% in SIRT1 protein expression levels. This increase suggests a possible activation of stress response pathways or tumor suppressor mechanisms in the model group, reflecting the underlying biological changes that occur under the experimental conditions. Overall, these results underscore the critical changes in various protein expressions that may be associated with the physiological responses observed in the study.

### 2.10. Transcriptome Sequencing

**Figure 7:**
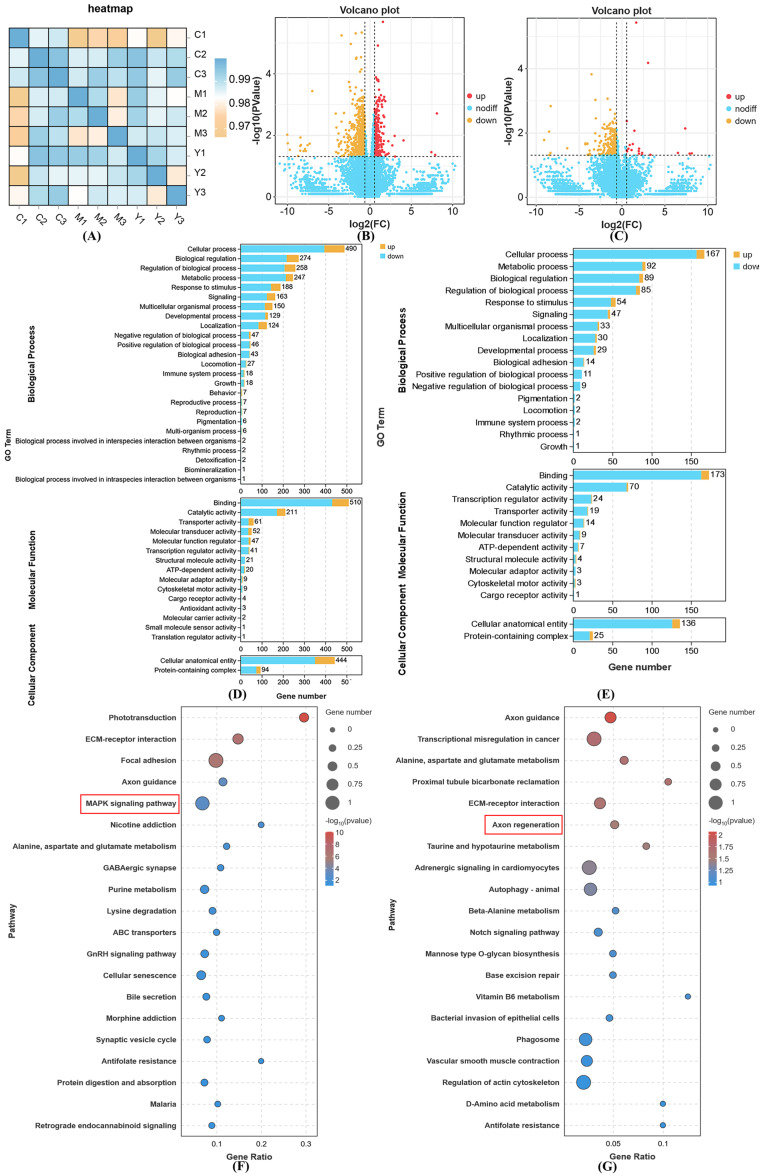
Effect of LRAs on gene expression during H_2_O_2_-induced zebrafish aging. (**A**) The correlation heat map between samples; (**B**) volcano map of DEGs in the C vs. M group; (**C**) volcano map of DEGs in the Y vs. M group; (**D**) GO annotation analysis of DEGs in the C vs. M group; (**E**) GO annotation analysis of DEGs in the Y vs. M group; (**F**) KEGG enrichment analysis of DEGs in the C vs. M group; (**G**) KEGG enrichment analysis of DEGs in the Y vs. M group.

The sequencing platform, Illumina NovaSeq X Plus, recorded a total data output of 59.74 GB. To maintain stringent data quality, post-filtration is essential; the Q20 metric should exceed 90%, the Q30 metric should surpass 85%, and the GC content must range from 40% to 50%. Adhering to these benchmarks confirms that there are no significant issues during sequencing, thereby ensuring the high reliability of the acquired data. Following the quality control process, the samples derived from 59.05 GB of valid data displayed a minimum Q20 value of 97.73%, while the lowest Q30 value noted was 93.73%. Moreover, the overall GC content fluctuated between 45% and 47%, reflecting an exceptional sequencing data quality and robust data reliability. The correlation among samples serves as a crucial metric for assessing the experimental reliability and the appropriateness of the sample selection. Correlation coefficients nearing 1 suggest a higher similarity in expression patterns between samples. We stipulate that the R^2^ between biological replicates should exceed 0.8; otherwise, careful interpretation of the sample is warranted, or the experiment must be repeated. A heatmap depicting the correlations among samples can be found in Figure 7A. The R^2^ values for most samples in the test group are above 0.9, indicating that the reproducibility among samples aligns with the requirements for further analysis.

Typically, genes that are differentially expressed are identified using criteria like fold change and significance levels. In this study, we defined the criteria for screening differentially expressed genes as |log2FC| ≥ 1.5 and *p <* 0.05. Figure 7B illustrates that there were 985 differentially expressed genes identified between group C and group M, with 217 genes being up-regulated and 768 down-regulated. Additionally, Figure 7C shows that there were 332 differential genes detected between the Y group and the M group, comprising 20 up-regulated genes and 312 down-regulated genes.

As shown in Figure 7D, the GO results show that compared with group M, a total of 49 items were screened out in group C. In BP, the most important entries are cellular process, biological regulation, the regulation of biological processes, metabolic processes and responses to stimulus. In MF, the most important entries are binding, catalytic activity, transporter activity, molecular transducer activity, and molecular function modulators. In CC, the most important entries are a cellular anatomical entity and protein-containing complex.

As shown in number 7E, the GO results show that compared with the M group, a total of 30 items were screened out in the Y group. In BP, the most important entries were cellular process, metabolic process, biological regulation, the regulation of biological process and responses to stimulus. In MF, the most important entries were binding, catalytic activity, transcription regulator activity, transporter activity, and molecular function regulator. In CC, the most important entries were cellular anatomical entity and protein-containing complexes.

As shown in Figure 7F, compared with group M, the pathways with a significantly enriched KEGG in group C are as follows: phototransduction, ECM–receptor interaction, focal adhesion, and axon guidance. As shown in Figure 7G, compared with group M, the pathways with a significantly enriched KEGG in group Y were as follows: axon guidance, transcriptional misregulation in cancer, alanine, aspartate and glutamate metabolism, proximal tubule bicarbonate reabsorption, ECM-receptor interaction, axon regeneration, taurine and hypotaurine metabolism, adrenergic signaling in cardiomyocytes, and animal autophagy. Through the GO and KEGG analysis results, we found that LRAs may affect transcriptional regulation, metabolism, nervous system function, cardiac regulation and other aspects to alleviate H_2_O_2_-induced aging. Moreover, SIRT1 was closely related to transcriptional regulation, metabolism, nervous system function and cardiac regulation, thus playing an important role in this through its deacetylation.

### 2.11. RT-PCR Results Correlated Strongly with RNA-seq Data

**Figure 8:**
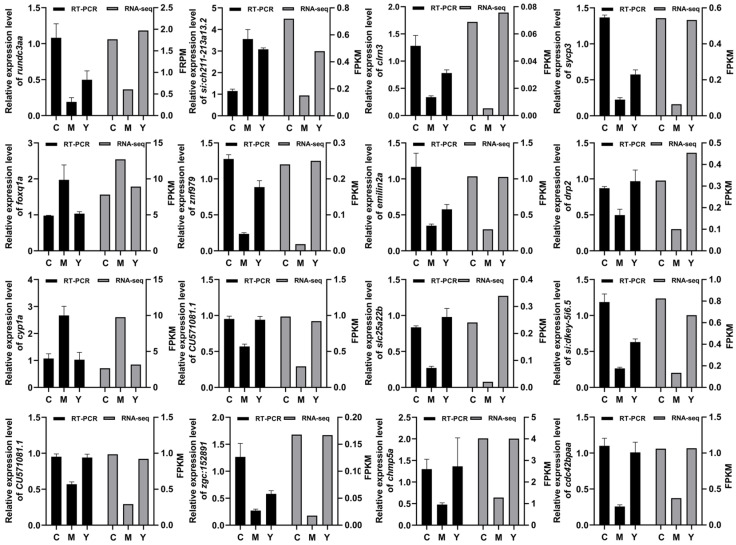
Validation of DEGs by RT-PCR. C represents Control. M represents the H_2_O_2_ group. Y represents the LRA group.

As an established method for validating gene expression, RT-PCR is widely employed to corroborate RNA-seq data. By systematically comparing the results from both techniques, we can confidently conclude that the expression changes observed in aging-related genes through RNA-seq are reproducible. This cross-validation not only strengthens the credibility of our research findings but also establishes a robust foundation for subsequent functional investigations into aging-related genes. To rigorously assess the reliability of the RNA-seq dataset, we identified and prioritized fifteen aging-related differentially expressed genes (DEGs) for experimental verification. Through RT-PCR experiments, we performed a quantitative analysis of their expression levels. The RT-PCR results aligned closely with the RNA-seq data (Figure 8), thereby corroborating the accuracy and reproducibility of the transcriptomic findings. Notably, among the fifteen aging-related DEGs, the expression patterns of the majority exhibited concordant trends in both RT-PCR and RNA-seq analyses, demonstrating the complementary roles and methodological consistency of these two approaches in aging research.

## 3. Discussion

Aging is an inevitable phenomenon in the body’s physiological processes, but this degeneration process contributes to the occurrence and development of many aging-related diseases. The identification of natural compounds with anti-aging properties holds translational potential for improving geriatric healthcare. *Lycium ruthenicum* Murray, a medicinal plant with established economic and ecological value [30], demonstrates particular promise. Our study systematically evaluated the *Lycium ruthenicum* anthocyanins (LRAs) in D-galactose-induced H9c2 senescence and H_2_O_2_-accelerated zebrafish aging models. Excessive ROS can damage the structure of biological macromolecules, including DNA, lipids, and proteins, thereby accelerating aging and threatening the health and survival of organisms [31,32,33]. In our study, an increase in ROS levels was observed in H9c2 cells after D-gal treatment. In contrast, LRA administration reduced the ROS levels in these cells, suggesting that LRAs may slow aging by clearing ROS and inhibiting oxidative stress. This aligns with established mechanisms where anthocyanins scavenge free radicals via phenolic hydroxyl groups [31,32,33].

SA-β-gal is a well-established marker enzyme used to study senescent cells [34]. It is commonly employed to detect cellular senescence, as its expression significantly increases in senescent cells and tissues [35]. After D-gal treatment, the SA-β-gal activity in H9c2 cells was significantly elevated. On the contrary, its activity was significantly reduced after treatment with *Lycium ruthenicum* Murray anthocyanins. These findings were further confirmed by the TUNEL assay, which showed that D-gal induced a significant increase in apoptosis in H9c2 cells, while LRA treatment reduced apoptosis.

To validate the in vitro findings, zebrafish were used as an in vivo model. Zebrafish are commonly used in biology, pharmacology, and toxicology research for various reasons, including their low maintenance costs, fast embryogenesis, high transparency, large brood size, and genomic similarity to humans [36,37,38,39,40]. After H_2_O_2_ treatment, zebrafish exhibited a significantly reduced survival rate, an increased heart rate, and elevated levels of ROS and SA-β-gal. AO is used to stain zebrafish in an overall live manner, with the fluorescence of apoptotic cells being brighter and the nucleus of the apoptotic cells becoming smaller, breaking into multiple block structures, and the membrane wrapping the fragmented nucleus and protruding from the cells [41]. On the surface, the nucleus was still green and there was no nucleolus. After H_2_O_2_ treatment, the AO expression level increased significantly. The results showed that LRAs significantly reduced the number of heart beats per minute, apoptosis, and the ROS expression levels induced by H_2_O_2_ in the zebrafish model, and improved its survival rate.

The accumulation of H_2_O_2_ in cells leads to osmotic pressure changes, metabolic disorders, increased intracellular ROS, and oxidative damage, ultimately accelerating aging and contributing to various pathological changes [42]. Aging levels can be indirectly assessed by measuring the antioxidant enzyme activity and oxidative damage-related products. As organisms age, the activity of antioxidant enzymes such as SOD, CAT, and GSH declines, reducing their ability to scavenge free radicals [43]. This results in uncontrolled free radical cascades and increased levels of malondialdehyde (MDA), a marker of lipid peroxidation [44]. SOD, MDA, CAT, and GSH play crucial roles in the aging process. SOD and CAT are the primary antioxidant enzymes responsible for scavenging harmful substances, such as free radicals and hydrogen peroxide, thereby preventing cellular damage. MDA serves as a marker for free radical damage, while GSH provides potent reducing power to protect cells from oxidative stress. The imbalance and decline of these substances during aging often accelerate the deterioration of cells and organs. Therefore, regulating the levels of these antioxidants can be beneficial in delaying the aging process. Our results showed that H_2_O_2_ treatment increased MDA levels and decreased SOD, CAT, and GSH levels in zebrafish. However, LRA pretreatment reduced MDA levels and increased SOD, CAT and GSH levels.

Genes such as *Bax*, *Bcl-2*, *P53*, *P21*, *SIRT1*, *TNF-α*, *NF-κB*, and *MAPK* are associated with aging. *Bax* prevents the release of cytochrome c by inhibiting mitochondrial membrane permeability, thereby maintaining the survival of senescent cells. *Bcl-2*, an anti-apoptotic protein, inhibits the function of *Bax*, ensuring cell survival. *P53* senses DNA damage and activates repair mechanisms, while *P21* induces cell cycle arrest by inhibiting cyclin-dependent kinase activity. The activation of *NF-κB* promotes the production of senescence-associated inflammatory factors, thereby enhancing senescence-associated phenotypes. *TNF-α* accelerates the aging process by activating the *NF-κB* signaling pathway, which promotes cellular inflammatory responses. Additionally, *MAPK* influences the progression of aging by regulating the release of cytokines, apoptosis, and cell cycle control. *P53* determines whether cells undergo apoptosis or arrest by regulating the balance between *P21* and *Bax*/*Bcl-2.* Furthermore, *TNF-α* and *NF-κB* play a central role in the inflammatory response, exacerbating the aging process through the *MAPK* pathway.

In our study, LRAs reduced the expression of *Bax*, *P53*, *P21*, *TNF-α*, *NF-κB*, and *MAPK* in zebrafish while increasing *SIRT1* and *Bcl-2* expression, thereby exerting an anti-aging effect. Silent information regulators (SIRTs), a family of nicotinamide adenine dinucleotide (NAD^+^)-dependent protein deacetylases, play a critical role in regulating cellular processes such as DNA damage repair, gene transcription, energy metabolism, stress response, and apoptosis [45,46,47,48]. The P53 tumor suppressor gene is a key regulator of the cell cycle, differentiation and apoptosis [49]. Recent studies have shown that Caveolin-1 (Cav-1) activates P53 by inhibiting SIRT, thereby inducing cellular senescence [50]. Additionally, SIRT3 inhibits pulmonary epithelial senescence and ferroptosis by deacetylating P53 at K320 (lysine 320) [51], a conserved post-translational modification site critical for regulating DNA damage responses. Aging is associated with decreased SIRT activity and excessive P53 activation. Activation of the SIRT/P53 signaling pathway can mitigate aging and play a crucial role in anti-aging process [52]. In this study, zebrafish in the model group exhibited lower SIRT1 expression, higher P53 expression, and increased P21 expression compared to the blank group, confirming the involvement of the SIRT1/P53 pathway in aging. LRA pretreatment increased SIRT1 expression, decreased P53 expression, and reduced P21 expression, consistent with previous research. These findings suggest that LRAs alleviate aging by regulating the SIRT1/P53 signaling pathway.

To sum up, LRAs effectively mitigate aging by activating the SIRT1/P53 signaling pathway. This provides a robust foundation for developing anti-aging products using *Lycium ruthenicum* Murray. Anthocyanins extracted from this medicinal plant could be formulated into functional foods, establishing a scientific basis for developing health-promoting products.

## 4. Materials and Methods

### 4.1. Materials and Chemicals

D-galactose (D-gal), a CCK-8 kit, DAPI kit and TUNEL kit were purchased from Sangon Bioengineering (Shanghai, China). *Lycium ruthenicum* Murray anthocyanins (LRAs) were provided by the Northwest Plateau Institute of Biology, Chinese Academy of Sciences (Xining, China). A reactive oxygen species (ROS) detection kit, acridine orange (AO) kit, senescence-associated β-galactosidase (SA-β-gal) staining kit, malondialdehyde (MDA) content detection kit, and catalase (CAT) activity detection kit were purchased from Biyuntian (Shanghai, China). A superoxide dismutase (SOD) activity detection kit and glutathione (GSH) content detection kit were purchased from Nanjing Jiancheng (Nanjing, China).

### 4.2. Cell Cultures

H9c2 rat cardiomyocytes were purchased from Fenghui Biotechnology (Changsha, China). Cells were maintained in DMEM medium (Sangon Biotechnology, Shanghai, China) supplemented with 10% fetal bovine serum (FBS, Sangon Biotechnology, Shanghai, China) at 37 °C in a 5% CO_2_ humidified incubator. Cells were passaged when the density reached 90%, with a passage ratio of 1:3.

### 4.3. Assessment of Cell Viability

H9c2 cells were seeded into 96-well plates at a density of 1 × 10^4^ cells/well and cultured for 24 h. The supernatant was carefully aspirated. The model group and treatment group were treated with 50 g/L D-gal, while the control group was treated with complete culture medium. After 24 h of treatment, the supernatant was carefully aspirated. In the treatment group, cells were exposed to a medium containing different concentration gradients of LRAs (200, 300, 400, 500, and 600 μg/mL). The control group and model group were supplemented with complete culture medium. Cells were further cultured for 24 h, followed by the addition of CCK-8 solution. After incubation at 37 °C in a 5% CO_2_ incubator for 30 min, the absorbance was measured at 450 nm using a microplate reader.

### 4.4. TUNEL Assay for Detecting Apoptosis in H9c2 Cells

H9c2 cells in the logarithmic growth phase were seeded in a 6-well plate at a density of 2 × 10^6^ for modeling and treatment. The cells were washed three times with PBS and then incubated with 4% paraformaldehyde for 30 min at room temperature. After washing three times with PBS, 0.3% TritonX-100 was added, and the cells were incubated at room temperature for 5 min. The cells were washed twice with PBS, and 50 μL of pre-prepared TUNEL detection solution (1:9:10) was added to each sample. The samples were incubated at room temperature in the dark for 60 min. After washing three times with PBS, DAPI solution was added for counterstaining, and the cells were incubated at room temperature in the dark for 5 min. The cells were washed three times with PBS, and an anti-fluorescence quenching solution was added for sealing. Cell apoptosis was observed under a fluorescence microscope (LSM 900, ZEISS, Oberkochen, Germany).

### 4.5. Maintenance and Treatment of Zebrafish

Wild-type zebrafish (AB strain) were purchased from Hunter Biotechnology, Inc. (Hangzhou, China; animal production license number: SCXK (Zhejiang) 2022-0003) and raised in a circulating reservoir system. The experiment was approved by the Medical Science Research Ethics Committee of Qinghai University School of Medicine (Approval No. 2022-18, 12 March 2022). One day before ovulation, zebrafish were placed in a spawning pool at a male-to-female ratio of 1:1. The plugboard was removed before light exposure the next day, and eggs were collected after 1 to 2 h of natural light. The collected eggs were cultured in embryo medium at a constant temperature of 28.5 °C. At approximately 8 h post-fertilization (8 hpf), embryos (n = 25) were transferred to 12-well plates containing embryo medium. Different concentrations of LRAs (250, 500, 1000 μg/mL) were added to the treatment group and incubated for 1 h. Then, 5 mM of H_2_O_2_ solution was added to the model group and treatment group, and incubation continued until 24 hpf. After incubation, the medium was replaced with fresh embryo medium, and incubation continued for 2 days. Finally, the survival rate and heart rate changes for each group were calculated. The 1-min heart rate was recorded in the atria and ventricles of zebrafish under a microscope.

### 4.6. Determination of ROS and Apoptosis Levels

DCF-DA (2′,7′-dichlorodihydrofluorescein diacetate) was used to detect ROS generation. The treated zebrafish or H9c2 cells were selected, and 500 μL of embryo water or culture medium containing DCFH-DA was added, followed by incubation at 37 °C in the dark for 20 min. They were then washed three times with embryo water or PBS. Acridine orange staining was used to detect cell death in live embryos. The treated zebrafish were selected, and 500 μL of embryo water containing acridine orange staining solution was added, followed by incubation at 28 °C in the dark for 20 min. They were then washed three times with embryo water. Before imaging, the zebrafish larvae were washed with embryo medium and anesthetized. Observations were made under a fluorescence microscope, and the fluorescence intensity was quantitatively analyzed using ImageJ (version 1.48V, NIH, Bethesda, MD, USA).

### 4.7. Determination of SA-β-gal Content

The treated zebrafish or H9c2 cells were selected, washed three times with embryo water or PBS, and 1 mL of β-galactosidase staining fixative was added. The samples were fixed overnight at 4 °C. The fixative solution was aspirated, and the samples were washed three times with embryo water or PBS. After removing the culture medium, 1 mL of staining working solution was added to each well and incubated overnight at 37 °C. Zebrafish were observed using a stereomicroscope (MZX100, Mshot, Guangzhou, China), and H9c2 cells were observed using an inverted fluorescence microscope and analyzed with ImageJ software (version 1.48V, NIH, Bethesda, MD, USA).

### 4.8. Biochemical Indicator Detection

According to the instructions of the SOD, MDA, CAT, and GSH kits, extraction solution was added to the processed zebrafish samples. The samples were subjected to ultrasonic disruption and centrifugation, and the supernatant was collected. The colorimetric method was used to measure the absorbance at wavelengths of 450 nm (SOD), 532 nm (MDA), 405 nm (GSH), and 520 nm (CAT) using a microplate reader (iMark680, Bio-Rad, Hercules, CA, USA). Results were calculated according to the kit instructions.

### 4.9. RNA-Seq and Data Analysis

For transcriptomic profiling, the blank group received no treatment (C group), the model group was treated with 5 mM of H_2_O_2_ (M group), and the treatment group was first treated with 5 mM of H_2_O_2_ followed by treatment with 1000 μg/mL LRAs (Y group) (n = 90 per group). Total RNA was extracted from treated zebrafish larvae using TrIzol reagent (YEASEN Biotechnology Co., Ltd., Shanghai, China). The concentration and purity of RNA were measured using a NanoDrop ND-1000 spectrophotometer (Thermo Fisher Scientific, Waltham, MA, USA). Furthermore, the integrity of the RNA was evaluated through 1% agarose gel electrophoresis, aided by a Universal Hood II transilluminator gel documentation system (BIO-RAD, Hercules, CA, USA). Library preparation and sequencing procedures were conducted at Gene Denovo Biotechnology Co. located in Guangzhou, China. Following these procedures, Gene Ontology (GO) function analysis and Kyoto Encyclopedia of Genes and Genomes (KEGG) pathway enrichment analyses were executed to thoroughly examine the primary functions of the differentially expressed genes (DEGs). These analyses aimed to identify pathways that were significantly enriched, providing insights into the biological processes and molecular functions associated with the identified DEGs.

### 4.10. Real-Time Quantitative PCR (RT-qPCR) Analysis

Total RNA was extracted from Zebrafish using TrIzol reagent. cDNA was synthesized using a reverse transcription kit (TaKaRa, Dalian, China). The reaction was performed on a real-time fluorescence quantitative PCR machine (Bio-Rad, Hercules, CA, USA) using a SYBR premixed Taq kit (TaKaRa, Dalian, China). The expression levels of target mRNA were calculated using the 2^−ΔΔCt^ method, normalized to β-actin. Primers were designed using Primer5 software (version 5.0V, Premier, Blainville, QC, Canada), and the sequences are listed in Table 1.

### 4.11. Western Blotting

Total protein was extracted using the protein extraction kit, and the protein concentration was determined using a BCA kit (Sangon, Shanghai, China). Proteins were separated by SDS-PAGE and transferred to a polyvinylidene fluoride (PVDF) membrane using a Bio-Rad wet transfer device (Hercules, CA, USA). After transfer, the PVDF membranes were blocked in blocking solution at 4 °C for 2 h. The membranes were then incubated with primary antibodies against SIRT1, P53, and P21 (Proteintech Co., Ltd., Shenzhen, China) overnight at 4 °C. After washing three times with TBST buffer (10 min each), the membranes were incubated with antibodies for 2 h at room temperature and washed three times with TBST (10 min each). Finally, an ECL solution (Beyotime, Shanghai, China) was prepared according to the kit instructions, and the membranes were developed using a chemiluminescence imaging system (Beyotime, Shanghai, China), and grayscale scanning analysis was performed.

### 4.12. Statistical Analysis

All data in this study were expressed as mean ± SD. Data were analyzed using SPSS version 26 (IBM Corp., Armonk, NY, USA) employing one-way analysis of variance (ANOVA) followed by least significant difference (LSD) post hoc tests. Differences between groups were considered statistically significant at *p*-value < 0.05, calculated by GraphPad Prism. Data visualization was performed using GraphPad Prism version 8.0.2 (GraphPad Software, San Diego, CA, USA).

## 5. Conclusions

This study demonstrates that LRAs have the potential to alleviate aging. LRAs mitigate aging by activating the SIRT1/P53 pathway. These findings can promote LRAs’ utilization, enhance their economic benefits, and support research on natural ingredients for anti-aging purposes.

## Figures and Tables

**Table 1:** Primer sequences used in this study.

Gene Name	Forward Primer (5′-3′)	Reverse Primer (5′-3′)
*Bax*	CGGCATGGCGACAGGGATG	CATAGCAGGAGACGGTGGTGATG
*Bcl-2*	CTCCTTCTCATACTTCAGCCTCCAC	ACCTTCAATGCCTCCTCCATCTTAC
*NF-κB*	CCTGTCTGTCTGTCTGTCTGTCTG	TCGTGGTGTCGTTGCTCTTCTC
*MAPK*	GTCCTACAGCAGCACAACTTCTAC	TCAACCCACAACGAAACACTCAG
*P21*	CCAGAGACGACACCGTTTATT	GGAAGACTGAGGAATGGATCTTT
*P53*	CGAGCCACTGCCATCTATAA	CTGATTGCCCTCCACTCTTATC
*SIRT1*	CGCAAAGACATCAACACGTTAG	CAGGAATCCCACAGGAAACA
*TNF-α*	AGGAGAGTTGCCTTTACCGC	AATGGATGGCAGCCTTGGAA
*foxq1a*	ACTCCATCGCTACGCCTCCTTC	CATACGGCAGCACAGTGTCCAC
*cyp1a*	CAGTGTCGTGTGGCTCTTCGTAG	TCGGTCTTCGCAGTGGTTGATAAG
*rundc3aa*	AGAGTTGCTGACATTGCGGAGAAG	TGGACGGTGGGTGGGTTATGG
*sycp3*	GCGGATCTGACGAAGACACGAG	AAACATCCCGACAGCACACAAGG
*zgc:152891*	AGACATGCGGTACAGGTGAATTGG	GCCCTTCCAGCCACAACTCATC
*chmp5a*	AAACAAGCGGCGTCTCCCAAC	AACACTCAGCATCCACAGCACATC
*slc25a22b*	ACTTCTGCCCTTTGCCCTTTGTC	TCTGCTTGCTTGTCTGGAACTCTC
*emilin2a*	AGAAGGACAGGAGAACGGCTACG	TTGGCGGTTTGTGGGCATGAG
*si:ch211-213a13.2*	GAACACGAGGTCAGGCAGATTCAG	GACTTCCCGATGCCAGCAACTC
*BX936382.1*	CAATGGTATCGGCTGGCGGAAC	GACGAGGAACGGAGTTTCTGGATG
*clrn3*	TTTCGGGAAGAGAGTGGGTGAGG	GAGGCTGCTGTTGACTGACTGAC
*drp2*	ATATTCGGCGGTTCAAGGCAAGG	TTGGCTGTCTCGTCTGTCGTTTG
*si:dkey-5i16.5*	ACTCCTTCCTGTACGGCTAACTGG	ATGCTCGCTCGCTGGAACATAAC
*cdc42bpaa*	CACCAGCACCAGGGACATAACAC	TGAGGATGAAGGAAGCAACAGCAG
*znf979*	ACTCCATAACCTCTGCTCCTCTGC	ACCTCCCTACCTCTCCTCCTCAC
*CU571081.1*	CCGCCGCAGAAGCTCAATGG	CGAGGGTCCACGAGAAGTTGTTTG
*β-actin*	TCGAGCAGGAGATGGGAACC	CTCGTGGATACCGCAAGATTC

## Data Availability

The original contributions presented in the study are included in the article. Further inquiries can be directed to the corresponding author.

## Abbreviations

List of Abbreviations			
LRA	*Lycium ruthenicum* Murray	H_2_O_2_	Hydrogen Peroxide
D-gal	D-galactose	GSH	Glutathione
ROS	Reactive Oxygen Species	DAPI	4′,6-diamidino-2-phenylindole
SA-β-gal	Senescence-Associated β-Galactosidase	CCK-8	Cell Counting Kit-8
TUNEL	Terminal Deoxynucleotidyl Transferase-mediated dUTP-biotin Nick End Labeling	ECL	Enhanced Chemiluminescence
RT-PCR	Reverse Transcription-Polymerase Chain Reaction	TBST	Tris-Borate-Sodium Tween-20
WB	Western Blotting	RNE-seq	RNA sequencing
AO	Acridine Orange	KEGG	Kyoto Encyclopedia of Genes and Genomes
MDA	Malondialdehyde	GO	Gene Ontology
CAT	Catalase	DEGs	Differential expressed genes
SDS-PAGE	Sodium Dodecyl Sulfate-Polyacrylamide Gel Electrophoresis	PVDF	Polyvinylidene Fluoride
